# Exploring short-term memory and listening effort in two-talker conversations: The influence of soft and moderate background noise

**DOI:** 10.1371/journal.pone.0318821

**Published:** 2025-02-06

**Authors:** Chinthusa Mohanathasan, Cosima A. Ermert, Janina Fels, Torsten W. Kuhlen, Sabine J. Schlittmeier

**Affiliations:** 1 Institute of Psychology, Work and Engineering Psychology, RWTH Aachen University, Aachen, North Rhine-Westphalia, Germany; 2 Institute for Hearing Technology and Acoustics, RWTH Aachen University, Aachen, North Rhine-Westphalia, Germany; 3 Visual Computing Institute, RWTH Aachen University, Aachen, North Rhine-Westphalia, Germany; Harvard Medical School, UNITED STATES OF AMERICA

## Abstract

Listening to conversations and remembering their content is a highly demanding task, especially in noisy environments. Previous research has mainly focused on short-term memory using simple cognitive tasks with unrelated words or digits. The present study investigates the listeners’ short-term memory and listening effort in conversations under different listening conditions, with and without soft or moderate noise. To this end, participants were administered a dual-task paradigm, including a primary listening task, in which conversations between two talkers were presented, and an unrelated secondary task. In Experiment 1, this secondary task was a visual number-judgment task, whereas in Experiments 2 and 3, it was a vibrotactile pattern recognition task. All experiments were conducted in a quiet environment or under continuous broadband noise. For the latter, the signal-to-noise ratio in Experiments 1 and 2 was +10 dB (soft-noise condition), while in Experiment 3 it was -3 dB (moderate-noise condition). In Experiments 1 and 2, short-term memory of running speech and listening effort were unaffected by soft-noise listening conditions. In Experiment 3, however, the moderate-noise listening condition impaired performance in the primary listening task, while performance in the vibrotactile secondary task was unaffected. This pattern of results could suggest that the moderate-noise listening condition, with a signal-to-noise ratio of -3 dB, required increased listening effort compared to the soft-noise and quiet listening conditions. These findings indicate that listening situations with moderate noise can reduce short-term memory of heard conversational content and increase listening effort, even when the speech signals remain highly intelligible.

## Introduction

Face-to-face conversations are probably the most prevalent and fundamental form of verbal communication used for sharing information. In the office, school, or restaurant, listening to conversations often has to be accomplished in settings where the speech signals of interest are accompanied by background noise. In such situations, the perceptual quality of the auditory signal might still be high enough for the target speech to remain highly intelligible. However, listening in these conditions can still be demanding. For example, for the immediate serial recall of heard but unrelated syllables, Surprenant [1] demonstrated that short-term memory performance can be reduced even when the speech signals are highly intelligible under different levels of noise (broadband noise presented at a signal-to-noise ratio (SNR) of +5 dB or +10 dB). Specifically, the addition of noise (+10 dB SNR) resulted in decreased short-term memory performance, and slightly more noise (+5 dB SNR) further reduced performance. The reduction in memory performance, even when speech is highly intelligible, was attributed to the strain on cognitive resources when listening to speech in noise (i.e., listening effort, which we address in more detail later). As a result, fewer resources were available for further cognitive processing of the speech content [1].

Research on listening, including that on listening effort, is dominated by studies using unrelated digits, letters, words, or isolated sentences with single-talker recordings [e.g., 1–8]. While such studies provide valuable insights, it is unclear whether the findings can be applied to more complex listening tasks, such as conversations between two talkers. Extracting, processing, and maintaining the semantic content of conversations requires a complex interplay of auditory processes and several basic cognitive functions, such as short-term memory, verbal-logical reasoning, and attention [9]. This is because the mental representation of the text and the eventual comprehension of the overall content need to be cyclically built up and revised during ongoing information reception (see the Construction-Integration Model, [10]). The present study examines the impact of soft- and moderate-noise or quiet listening conditions, in all of which speech intelligibility is high, on memory and listening effort related to conversations between two talkers.

Fintor et al. [11] addressed a research gap by investigating listeners’ short-term memory in a listening task that was more complex and closer to real life (cp. [12,13]). In two experiments, memory for running speech was measured by asking participants to answer content-related questions immediately after listening to a two-talker conversation in quiet conditions. The study investigated the effect of spatial separation versus co-location of two conversing talkers on the listeners’ memory and cognitive spare capacity using a dual-task paradigm. In Experiment 1, a visual number-judgment task was assessed as a secondary task, and in Experiment 2, this number-judgment task was combined with a visual letter-judgment task. Although memory performance was similar regardless of whether the two talkers were acoustically co-located or spatially separated, a performance benefit in the secondary tasks was observed in the latter condition. This indicated that auditory spatial separation of the talkers was less straining on cognitive resources in the primary listening task, thereby leaving more resources available for the secondary task. Even in quiet conditions, variations in cognitive spare capacity were observed, indicating variations in listening effort [11].

In the Framework for Understanding Effortful Listening (FUEL), Pichora-Fuller et al. [14] define effort as “the deliberate allocation of mental resources to overcome obstacles in goal pursuit when carrying out a task” (p. S10), with listening effort referring specifically to listening tasks. When the same listening task is performed under different listening conditions, it may be possible to achieve comparable performance in the listening task, while a different degree of listening effort is required to achieve the observed level of performance [7,11]. Research on listening effort has exploited various methodologies (for a comprehensive review, see [15]). Dual-task paradigms were employed in the present study to assess both memory and listening effort in conversations involving two talkers, as in Fintor et al. [11]. In a dual-task paradigm, participants are instructed to perform two separate tasks in parallel, a primary and a secondary task. The dual-task paradigm exploits the fact that a person’s processing resources are limited [16]. Thus, the more resource-demanding the primary task, the fewer processing resources are available for the secondary task. The primary task employed in listening research is typically designed to directly measure listening-related performance, such as speech recognition [e.g., 7,17], isolated word recognition [e.g., 2,18], or, in the present study, remembering the conversational content. The secondary task is preferably independent of the listening task [19].

The dual-task paradigm can be used to compare listening conditions A and B, with certain resulting performance patterns serving as indicators of differences in listening effort. A performance pattern, that is commonly interpreted as a clear indication of higher listening effort under condition B, is when lower secondary task performance is observed under condition B than A while listening task performance is similar across both conditions. This suggests that under condition B the primary listening task required more cognitive resources, leaving fewer resources available for the secondary task [11,15,20]. Conversely, if the performance patterns in both the listening task and the secondary task remain constant across conditions A and B, it is considered that the listening effort exerted was similar in both conditions. A clear indication of a higher burden on cognitive processing resources in condition B and thus higher listening effort can be inferred in two other instances, namely: (a) when the listening task performance is lower in condition B than in A, yet the secondary task performance remains similar in both conditions, or (b) when a decline in both listening and secondary task performances in condition B is observed. In contrast, there is no clear indication for or against variations in listening effort if, in one condition, performance in the listening task is reduced while performance in the secondary task is increased compared to the other listening condition. This pattern could indicate a shift in strategy from one listening condition to another, e.g., participants prioritized the secondary task over the listening task rather than a change in listening effort.

The secondary tasks that have been used in dual-task studies of listening effort are quite diverse, ranging from responding to visual probes [e.g., 18,21] to recognizing a vibrotactile pattern [e.g., 17,22]. The choice of the secondary task can impact the sensitivity of a paradigm to detect changes in listening effort between two conditions. If a secondary task is relatively easy and requires only few cognitive resources, it is possible that changes in listening effort cannot be detected within the dual-task paradigm [4]. Accordingly, a more demanding secondary task could improve a paradigm’s sensitivity to reveal variations in listening effort between different listening conditions. Picou and Ricketts [4] argued that a vibrotactile secondary task is more demanding than a secondary task where participants have to respond to a simple visual probe. This is because a vibrotactile secondary task necessitates more cognitive resources.

Even when speech intelligibility in conversations remains high in noisy listening conditions, more cognitive resources might be needed to understand and remember speech [1]. This leads to a decrease in available resources for additional cognitive processing, such as performing a secondary task. Under soft or moderate noise conditions, where speech intelligibility is high, understanding and remembering heard conversational content may require more cognitive resources and listening effort compared to quiet conditions.

Such effects would be consistent with the Ease of Language Understanding model [the ELU model, 23]. The ELU model provides a conceptual framework for understanding the interplay of speech comprehension, listening effort, and auditory-perceptual information. In a nutshell, the ELU model differentiates between two cognitive processing routes for speech understanding, namely an implicit and an explicit route. The explicit route is activated when the implicit route fails, which may occur when there is a mismatch between the mental representation of an auditory speech signal and its long-term memory representation due to the signal being degraded or distorted. In comparison to the implicit route, the explicit route is slower, more focused, and more resource-intensive. The more explicit processing is required, the higher the listening effort. The ELU model can also be applied to the potential role of auditory-perceptive information in memory functioning in a quiet or soft- and moderate-noise listening condition [11].

### Research intent

The present study examines the role of soft- and moderate-noise or quiet listening conditions on a complex listening task, namely comprehension and memory of two-talker conversations, and listening effort. In three experiments, the effects of continuous broadband noise on memory for speech content and listening effort were investigated using a dual-task paradigm. For this purpose, participants were administered a primary listening task in which a coherent text, which was spoken by two talkers taking turns, was presented and corresponding questions assessing memory and comprehension had to be answered [11,24]. These conversations were presented binaurally via headphones at 60 dB(A) in soft- or moderate-noise conditions (continuous broadband noise) or quiet conditions. The SNR of the continuous broadband noise was set to +10 dB (soft-noise condition) in Experiment 1 and Experiment 2, and to -3 dB (moderate-noise condition) in Experiment 3. In Experiment 1, we used a visual number-judgment task as a secondary task that was performed concurrently with the primary listening task. This task has previously been used successfully in quiet conditions to reveal the role of auditory spatial cues for effortless listening to a two-talker conversation [11]. In Experiment 2 and 3, we used the same primary listening task as in Experiment 1, but with a vibrotactile secondary task [22]. This task is more demanding and may be more sensitive to changes in listening effort.

We argue that listening conditions with soft and moderate noise, where speech remains highly intelligible, drive the activation of the explicit processing route in the ELU model [23], thus leaving fewer processing resources for other cognitive tasks. This is because soft- and moderate-noise listening situations might involve more focused attention and cognitive processing to comprehend speech and memorize the conversational content compared to quiet listening situations.

Our experiments explored two main hypotheses. Firstly, we tested whether participants would show more errors and/or slower reaction times in the vibrotactile pattern recognition task than in the visual number-judgment task. Secondly, we investigated whether participants’ short-term memory performance is poorer and/or their listening effort is higher in two-talker conversations accompanied by soft and moderate broadband noise, where speech is highly intelligible, compared to two-talker conversations in quiet conditions.

## Methods Experiments 1–3

### Ethical approval

The research has complied with all relevant national regulations, institutional policies, and by the tenets of the Helsinki Declaration. The research was pre-approved by the local ethics committee at the Philosophical Faculty of the RWTH Aachen University (“Listening to and remembering conversations between two talkers: Cognitive research using embodied conversational agents in audiovisual virtual environments”, 2021_08_FB7_RWTH AACHEN).

### Participants

50 participants took part in Experiment 1. Due to technical difficulties in six sessions and two participants having impaired hearing, as assessed by a hearing screening, the final sample *n*_*1*_ included 42 participants (29 female, 12 male, 1 non-binary), aged between 18 and 62 years (*M* = 24.9 years, *SD* = 9.1).

A new sample of 39 participants was examined for Experiment 2, from which three datasets were discarded because of technical difficulties during two experiments and because one participant showed impaired hearing as assessed by hearing screening. This resulted in a total of *n*_*2*_ = 36 participants (28 females and 8 males), aged 18 to 43 years (*M* = 22.5 years, *SD* = 4.7).

In Experiment 3, 41 new participants initially participated. Following the exclusion of two due to technical difficulties, the final sample size *n*_*3*_ was 39 participants (26 females and 13 males), aged 18 to 43 years (*M* = 24.3 years, *SD* = 5.6).

Participants from all three experiments reported their vision as being normal or corrected to normal vision, and they were native German speakers. As assessed via pulsed pure-tone ascending hearing screening (AURITEC Ear 3.0 with Sennheiser HAD 280 headphones), these participants had normal hearing sensitivity in both ears (<20 dB HL in the frequency range between 250 and 4000 Hz) [25]. Participants were recruited through email and received either course credit points or €10 for their participation. Written informed consent was obtained from all participants before the experiment started.

### Stimuli, instruments, and apparatus

All experiments were programmed in PsychoPy 2021.2.3 (Python 3.6.6; [26]) and ran entirely on a Dell Latitude 3590 laptop. All visual material was displayed on the laptop’s non-glare 15” screen, and all auditory stimuli were delivered via an external sound card (Focusrite Scarlett 2i2 2^nd^ Gen) and headphones (Sennheiser HD650). Vibrotactile stimuli (in Experiments 2 and 3) were presented using a Sony Dualshock 4 controller.

The speech signals were calibrated to a sound pressure level of 60 dB(A). The broadband noise (pink noise) was calibrated to 50 dB(A) in Experiments 1 and 2, and to 63 dB(A) in Experiment 3. The calibrations were conducted at the ear canal entrance of the artificial head developed by the Institute of Hearing Technology and Acoustics [27]. For Experiment 3, a pre-test (*n* = 6) was performed to determine the specified sound pressure level that would not impede speech intelligibility. Here, participants listened to short sentences from the AuViST database [28], which were also used in the current experiments. The sentences were presented with broadband noise presented at three different SNRs (+1 dB, -3 dB, and -7 dB). We played six sentences per SNR, one after the other, and participants were instructed to repeat each sentence after hearing it once. The lowest SNR, at which participants were able to recall the whole sentence correctly, was selected (-3 dB).

### Primary listening task

The primary listening task in all three experiments involved the Heard Text Recall (HTR) task presented audio-only [see 11,24,29]. The auditory stimuli were spoken coherent texts, each describing three generations of a family (grandparents, parents, and children), considering different aspects such as profession, hobbies, and age of the family members, as well as their relationship with each other. In each text, 5–6 people were mentioned by name. Names, hobbies, places, and ages differed in various texts, and particular care was taken to ensure that similarities were not too high either.

All speech material was obtained from the AuViST database [28] and is constituted of German texts [24] spoken by a female and a male speaker. The fundamental frequency of the male speaker was 120 Hz, and that of the female speaker was 175 Hz.

Each text consisted of ten sentences, and it was presented as a conversation between one talker with a female voice and another talker with a male voice. The turn-taking between the female and the male speaker aimed to simulate a natural conversation, so sentences linked closely together were spoken by the same conversational partner (the number of sentences spoken by the two conversational partners was counterbalanced). The conversational partners never spoke simultaneously to avoid, for example, partial masking of speech signals. There was a pause of 0.6 seconds between each sentence. The auditory stimuli were presented to both ears via headphones.

**Fig 1:**
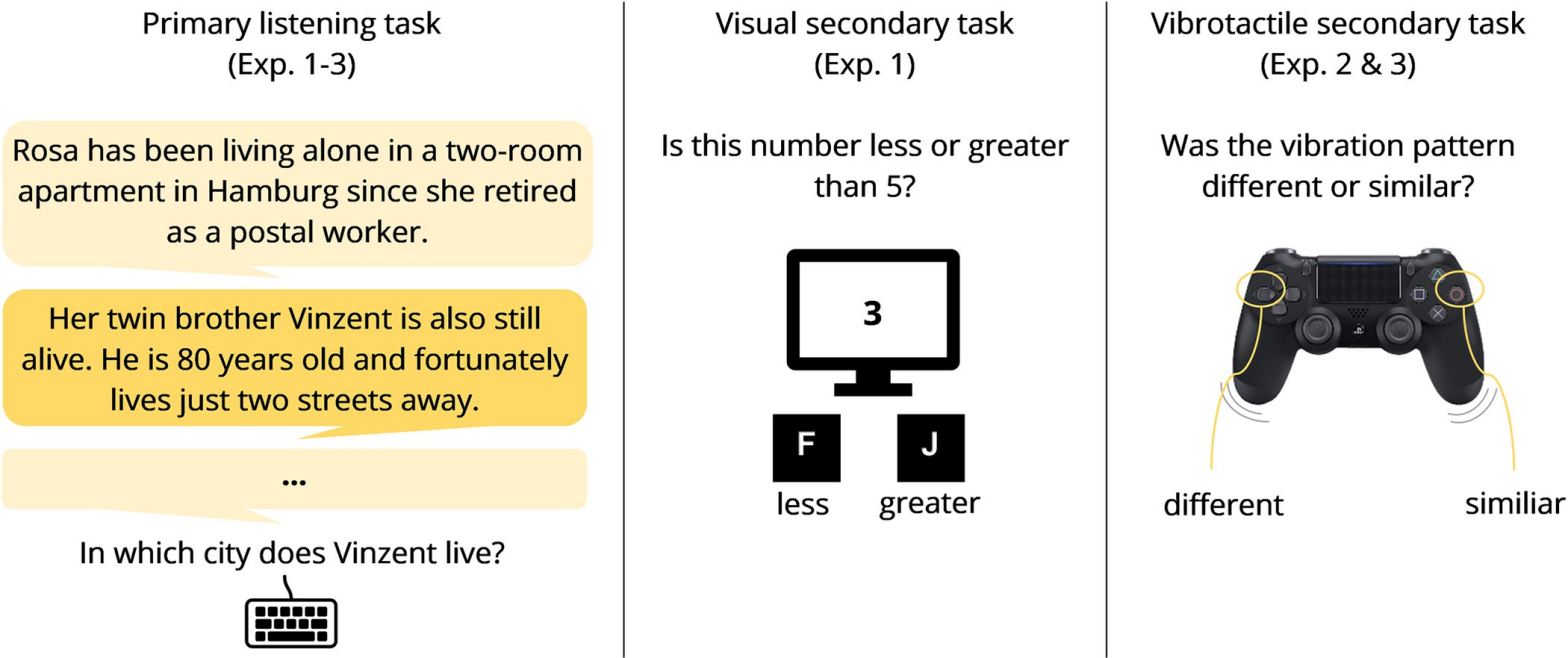
Overview of the primary and secondary tasks. The primary listening task was presented through headphones at 60 dB(A). In the visual secondary task, the numbers from 1–9, apart from 5, were presented. In the vibrotactile secondary task, two similar (short-short or long-long) or two different (short-long or long-short) vibration patterns were presented. If background noise was present, it was presented with an SNR of +10 dB (Experiments 1 and 2) or -3 dB (Experiment 3). Adapted from [12].

For each text, participants were required to answer nine corresponding questions presented on a computer screen. The questions asked for names of family members, relations between family members, and further information (e.g., profession, locations, hobbies, age). Questions related to one specific text were arranged in a fixed order but did not follow the order of the information from the conversational content. For example, the name of the mother could be told in the sixth sentence but asked back in the third question. Each question remained on screen until a response was given. Participants typed their answers using a keyboard. The participants could omit a question by pressing the spacebar and then the return button, but going back to a missed question or correcting a response after confirming the answer was not possible. The responses to each question could be provided in one or two words. Participants’ responses were manually scored afterward. Sample sentences and a sample question translated into English are shown in Fig 1. See Fig 1 for examples of sentences and questions in English translation.

### Visual secondary task

**Fig 2:**
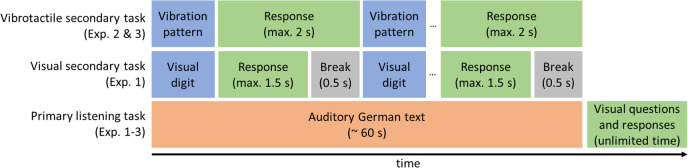
Timing structure of the primary and secondary tasks. The German texts in the primary listening task were presented through headphones, followed by questions presented on the laptop. Responses had to be typed on a keyboard. In the visual secondary task, digits from 1–9, except 5, were presented visually and participants had to respond whether the digit was less than or greater than 5. Responses were made using the two keys on the keyboard. In the vibrotactile secondary task, similar (short-short or long-long) or different (short-long or long-short) vibration patterns were presented via a game controller and participants had to identify whether the patterns were similar or different using two buttons on the controller.

The number-judgment task was used as a visual secondary task in Experiment 1 [11,30]. Here, black digits from 1 to 9, excluding 5, were displayed on a white background with a width and height of 1.5 cm in the center of the notebook screen. The viewing distance was about 60 cm. The viewing angle was about 84 arcminutes [31,32]. Each trial in the number-judgment task started with the onset of the visual stimulus and lasted until a response was made or 1500 ms had elapsed after the onset of the visual stimulus (see Fig 2). If a response was made, the next digit was presented after 500 ms. Participants were asked to indicate whether the presented digit was less than or greater than five. Responses were given by pressing the ‘f’ (less than five) or ‘j’ (greater than five) keys on a German keyboard with the left or right index finger, respectively.

### Vibrotactile secondary task

The vibrotactile pattern-recognition task was used as a vibrotactile secondary task in Experiments 2 and 3. In the vibrotactile pattern-recognition task, participants held a controller (Sony Dualshock 4) with both hands. The controller emanated four vibration patterns (short-short, long-long, short-long, and long-short) in the strong vibration spectrum. Participants were required to identify whether the two stimuli of a tactile pattern were similar (e.g., short-short) or different (e.g., short-long) by clicking the circle (O) or left arrow (PL) button on the game controller (see Fig 1).

A pre-test (*n* = 12), in which four versions of the task were tested, ensured that the version described and used in this study was neither too easy nor too difficult. In this pre-test, participants were presented with four vibrotactile patterns (short-short, long-long, short-long, or long-short). In one version of the task, participants had to categorize each vibrotactile pattern using four buttons (e.g., L1 for short-short, R1 for long-long, O for short-long, and PL for long-short). Within another version, they had to identify whether the tactile pattern was similar or different using two buttons (e.g., O for similar, so for both short-short and long-long). Since the game controller features two vibration motors, a stronger and a finer, less perceptible one, each version of the task had to be performed and tested under each setting.

The parameters to be set (length and level) to achieve the appropriate vibration stimuli were determined beforehand in a series of measurements using the Samsung Galaxy S6’s acceleration sensor and the Phyphox app [33]. The mobile phone was mechanically connected to the controller, and both were placed on a soft, non-springy surface. The Phyphox app was developed at RWTH Aachen University and is designed for conducting experiments with highly sensitive mobile phone sensors in a research and teaching context. We started with the same durations of the vibrotactile pattern recognition task as Gosselin & Gagné [22], where “short” was 250 ms and “long” was 500 ms separated with a pause of 300 ms.

These measurements indicated that the vibration pulses lasted longer than set and that the amplitude fluctuated by up to 50%. The adjustable pulse duration and level were then optimized so that the measured pulses corresponded to the actual requirements. Unwanted effects with short pulses affecting the amplitude or small delays changing the length of the pulses again were considered.

The final parameters of the tactile patterns used in the current task version are shown in Table 1. There was a pause of 0.3 s between the two vibrations that make up a vibration pattern. A constant response time of 2 s was used between the tactile patterns (see Fig 2). Thus, the number of tactile patterns, ranging from 19 to 22, for each text varied based on the length of the HTR sound files.

**Table 1:** Parameters of the vibrotactile secondary task using the Sony DualShock 4 controller.

	Vibration pattern			
**Parameters**	short-short	long-long	short-long	long-short
First Pulse (ms)	150	500	200	500
Second Pulse (ms)	100	350	400	100
First Level	0.9	0.4	0.7	0.4
Second Level	0.6	0.4	0.35	0.65

*Note*. To obtain similar parameters (short = 250 ms and long = 500 ms) as Gosselin & Gagné [22] used with the game controller, the vibration times, and the respective vibration levels were adjusted using the Samsung Galaxy S6’s acceleration sensor and the Phyphox app [33]. These measurements resulted in the parameters above. There is a pause of 300 ms between the two vibrations that make up a vibration pattern. In this study, the controller’s stronger instead of the finer, less perceptible, motor was used.

### Sociodemographic data

On the computer, all participants answered sociodemographic questions about their age, gender, mother tongue, eyesight, and handedness.

### Procedure

The experiments took place between March 3, 2022, and May 20, 2022 (Experiment 1), April 19, 2022 and May 31, 2022 (Experiment 2), and April 25, 2023 and May 12, 2023 (Experiment 3). All three experiments were conducted in individual sessions within a soundproof booth (Studiobox, premium edition) located within the Teaching and Research Area Work and Engineering Psychology of the RWTH Aachen University. Upon arrival, participants were provided with comprehensive briefing on the components of the corresponding experiment and the tasks in written form. Once the written informed consent form was signed, a hearing screening was conducted on all participants to ensure normal hearing. They then took part in the experiment, which lasted around 60 minutes.

At the beginning of each experiment, written instructions appeared on the screen, followed by a practice block to ensure that the participants were familiar with the tasks. Participants began with one single-task block of the primary listening task. This block included one text that was spoken by two conversational partners. It was followed by 20 practice trials of the secondary task (the visual number-judgment task in Experiment 1 and the vibrotactile pattern recognition task in Experiments 2 and 3). After these two single-task conditions, one dual-task block followed, in which the primary listening task and the secondary task had to be performed simultaneously. Participants were told at the start of each experiment that they should try to respond quickly and accurately to both tasks. In Experiment 3, where we used a vibrotactile secondary task, there was an addition in the practice session. Before practicing the vibrotactile secondary task, participants were allowed to acquaint themselves with the game controller, the tactile patterns, and the corresponding buttons they had to press. Practice on all tasks was conducted without any noise. However, after practicing single- and dual-tasking, participants could listen to the continuous broadband noise for as long as they liked to familiarize themselves with the sound and not be surprised by it in the test session.

Following the practice block, all the participants completed a counterbalanced (across participants) order of three conditions to reduce the possibility of confounding seriation and position effects. The conditions were: 1) a single primary listening task; 2) a single visual or vibrotactile secondary task; and 3) a dual-task (primary listening task and visual or vibrotactile secondary task concurrently). For the single listening task condition, participants responded to two texts. The single visual or vibrotactile secondary task condition consisted of 40 trials. In the dual-task conditions, six texts were presented (the text order was randomized across participants). Here, the number of trials of the visual or vibrotactile secondary task was defined by the duration of the listening task. After each text, the corresponding questions were presented on the screen one after the other, and the participants entered their responses via the laptop’s keyboard.

All tasks were carried out in a quiet condition and with broadband noise (condition order was counterbalanced over participants). A variable resting time was encouraged between the two blocks. In addition, in Experiment 3, each participant was asked verbally at the end whether the texts were intelligible, to which all participants responded in the affirmative. The experiment was concluded with a debriefing.

### Data analysis

The data analysis was conducted using R version 4.1.2 [34]. The dependent variables included performance measures in the HTR task (a binary variable indicating correct responses to each of nine questions per text in the primary task, with 1 representing a correct response and 0 representing an incorrect response), performance measures in the secondary task (a binary variable indicating correctness in the number judgment or vibrotactile pattern recognition, with 1 representing the correct responses and 0 representing the incorrect responses), and reaction times (RTs) of correct trials in the secondary task (measuring the duration between the appearance of the number, respective vibrotactile pattern, and the participant’s response).

The performance and RT data were analyzed using Generalized Linear Mixed-Effect Models (GLMMs) with the lme4 package (version 1.1.32) in R (see [35]). GLMMs allow for the modeling of individual-level variability and dependencies among observations. They are more flexible than ANOVA in handling mixed effects or non-normal data structures [36,37], often providing increased statistical power. Additionally, GLMMs do not require data transformation to yield a normal distribution, as they can directly model non-normal distributions using appropriate link functions. This flexibility is particularly useful for binary and RT data, often characterized by positive skewness and continuity.

The RT data modeling was conducted exclusively on correct responses, with incorrect or missing responses being excluded from the analysis. In accordance with the recommendations of Whelan et al. [38], RTs below 150 ms were identified as outliers and excluded from the analysis. Furthermore, RTs exceeding 2 *SD*s from the mean were identified as outliers and removed from the analysis, following the procedure of Berger and Kiefer [39]. In total, 5.10% of the RT data were removed. These outliers may reflect the rapid guessing or inattention of the participants. The performance data were modeled using GLMM with a binomial distribution and the logit link function, while the RT data were modeled using a gamma distribution and the log link function.

Three individual GLMMs were constructed to investigate the effect of *secondary task* and *acoustic condition* a) on performance in the primary task (HTR), b) on performance in the secondary task (visual number-judgment task or vibrotactile pattern-recognition task), and c) on RTs in the secondary task. For all GLMMs, we included random intercept to account for variability at the levels of participant, age, question, and/or text (see below). These random intercepts were modeled as independent, and thus the default diagonal covariance structure was employed. This approach avoids overfitting, particularly given the lack of theoretical justification for correlations between random effects (e.g., between participants and text). For each individual GLMM, the best-fitting model was determined by backward model selection. However, we conducted model optimization only for random (intercept) factors, not for fixed factors. Likelihood Ratio tests were used to compare models. Post hoc pairwise comparisons were performed based on estimated marginal means (emmeans) utilizing the emmeans package (version 1.10.2, see [40]).

- First, we investigated the effect of *acoustic condition* and *secondary task* on memory performance in the primary listening task across Experiments 1–3. For the GLMM analysis of memory performance, the following fixed factors were considered: *acoustic condition* (quiet in Experiments 1–3, soft background noise in Experiments 1 and 2, or moderate background noise in Experiment 3), *secondary task* (visual secondary task in Experiment 1 or vibrotactile secondary task in Experiments 2 and 3), *number of tasks* (listening task as a single task or as a dual task), the interaction of *acoustic condition* x *number of tasks*, as well as the interaction of *acoustic condition* x *secondary task*. The random (intercept) factors included *participant*, *age*, *question* (referring to each question in the primary listening task), and *text* (referring to each individual text). *Question* and *text* were included as random (intercept) factors to account for variability in difficulty. In the best-fitting model analyzing performance in the primary listening task, all initially considered factors were retained.
- Next, we investigated the effect of *acoustic condition* and *secondary task* on performance in the secondary task across Experiments 1–3. For the GLMM analysis of secondary task performance, we considered *acoustic conditions* (quiet in Experiments 1–3, soft background noise in Experiments 1 and 2, or moderate background noise in Experiment 3), *secondary task* (visual secondary task in Experiment 1 or vibrotactile secondary task in Experiments 2 and 3), *number of tasks* (secondary task as a single task or as a dual task), and their interactions as fixed factors. Furthermore, *participant* and *age* were included as random (intercept) factors. In the best-fitting model analysis of performance in the secondary tasks, all initially considered factors, except *age* were retained.
- Finally, we examined the effect of *acoustic condition* and *secondary task* on RTs in the secondary task across Experiments 1–3. For the GLMM analysis of secondary task RTs, the same fixed and random (intercept) factors were considered as for the model examining secondary task performance. As with secondary task performance, in the best-fitting model analysis of secondary task RTs all the factors originally considered were retained, apart from *age*.

## Results

### Performance in the primary task

**Fig 3:**
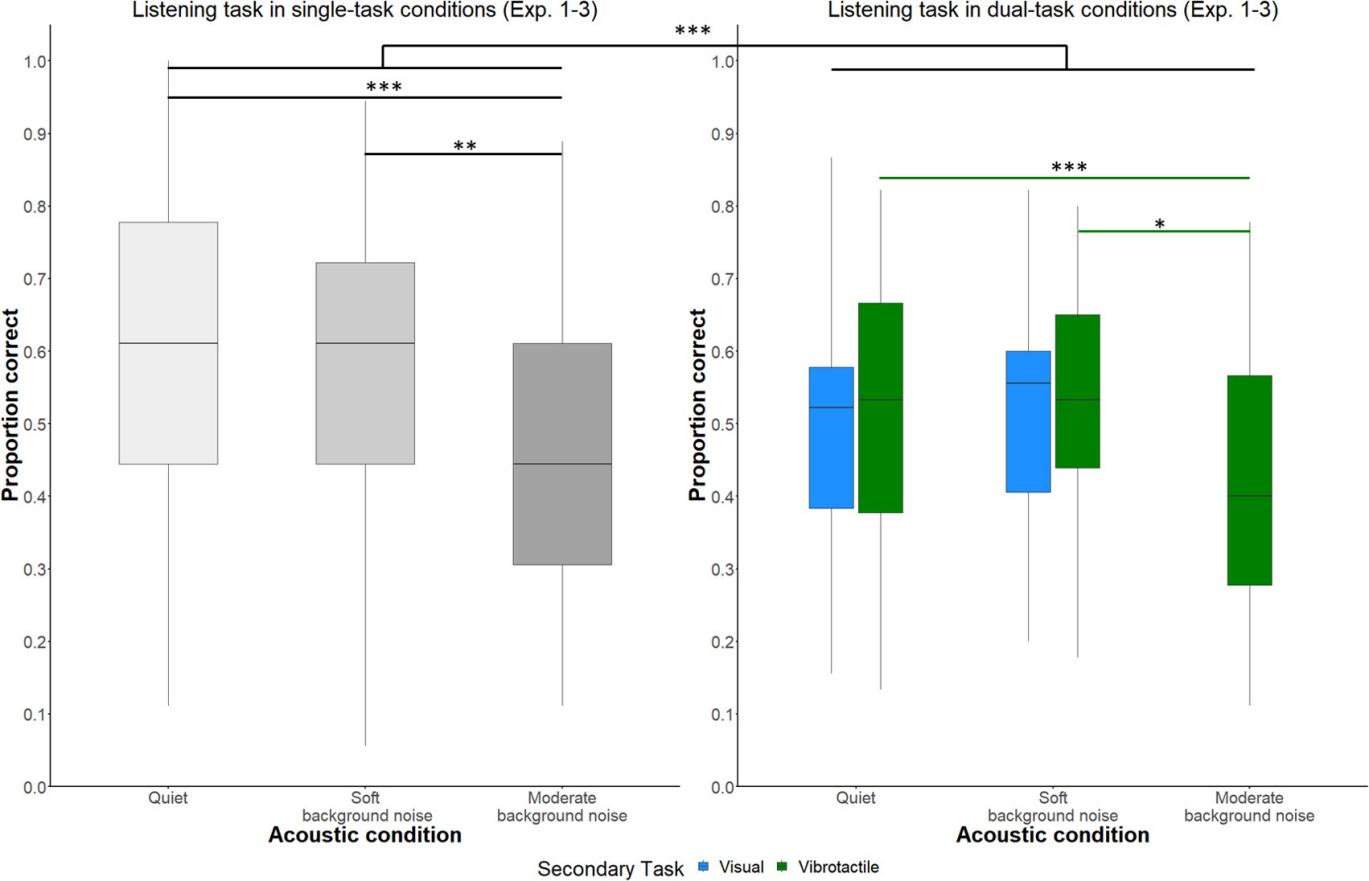
Performance in the primary listening task across Experiments 1–3. Left: Listening task performance in single-task conditions as a function of *acoustic condition* (quiet, soft background noise, moderate background noise). Right: Listening task performance in dual-task conditions as a function of *acoustic condition* and *secondary task* (visual number-judgment task, vibrotactile pattern recognition task). The boxplots show the data distributions for primary task performance (proportion correct). The boxes represent the interquartile ranges, while the lines contained within the boxes represent the medians. ***p < .001, **p < .01, *p < .05.

First, the effect of *acoustic condition* and *secondary task* on memory for conversational content was investigated based on performance in the primary listening task across all three experiments. The best-fitting GLMM consisted of *acoustic condition* (quiet in Experiments 1–3, soft background noise in Experiments 1 and 2, or moderate background noise in Experiment 3), *secondary task* (visual number-judgment task in Experiment 1 or vibrotactile pattern recognition task in Experiments 2 and 3), and *number of tasks* (listening task as a single task or as a dual task) and two-way interactions as fixed effects. *Participant*, *age*, *question*, and *text* were included as random (intercept) factors. The descriptive results are shown in Fig 3. Table 2 provides a summary of the final GLMM that modeled memory performance.

**Table 2:** Results from the final GLMM modeling performance in the HTR task as predicted by acoustic condition and secondary task.

Fixed effects	Estimate	*SE*	*z*	95% CI	*p*
Intercept	0.25	0.21	1.20	0.85, 1.94	0.23
Acoustic condition^a^					
Quiet	Reference				
Soft background noise	-0.01	0.08	-0.17	0.85, 1.15	0.86
Moderate background noise	-0.40	0.10	-3.77	0.55, 0.83	< .001
Soft background noise	Reference				
Moderate background noise	-0.38	0.11	-3.23	0.54, 0.86	< .001
Secondary task^b^					
Visual number-judgment task	Reference				
Vibrotactile pattern recognition task	0.15	0.09	1.67	0.97, 1.39	0.09
Number of tasks					
Single-task condition	Reference				
Dual-task condition	-0.46	0.08	-5.66	0.53, 0.74	< .001
Acoustic condition^a^ x Number of tasks					
Quiet x Single task	Reference				
Soft background noise x dual task	0.06	0.11	0.55	0.86, 1.30	0.58
Moderate background noise x dual task	0.03	0.12	0.28	0.82, 1.31	0.78
Quiet x Dual task	Reference				
Soft background noise x Single task	-0.06	0.11	-0.55	0.77, 1.16	0.58
Moderate background noise x Single task	-0.03	0.12	-0.28	0.77, 1.22	0.78
Soft background noise x Single task	Reference				
Moderate background noise x Dual task	0.14	0.13	1.03	0.88, 1.49	0.30
Soft background noise x Dual task	Reference				
Moderate background noise x Single task	0.03	0.15	1.67	0.77, 1.36	0.86
Acoustic condition^a^ x Secondary task^b^					
Quiet x Visual number-judgment task	Reference				
Soft background noise x Vibrotactile pattern recognition task	-0.16	0.10	-1.60	0.69, 1.04	0.11
Quiet x Vibrotactile secondary task	Reference				
Soft background noise x Visual secondary task	0.16	0.10	1.60	0.96, 1.43	0.11

*Note*. Number of observations: 14742; groups: Participant = 117, questions = 9, texts = 16. Confidence intervals calculated using the Wald method. Model equation: Performance ~ acoustic condition + number of tasks + secondary task + acoustic condition: Number of tasks + acoustic condition: Secondary task + (1|participant) + (1|age) + (1|question) + (1|text); family = binomial, link function = logit.

^a^ The primary listening task was presented in quiet (Experiments 1–3), in soft broadband noise (Experiments 1 and 2), and in moderate broadband noise (Experiment 3).

^b^ The secondary task was a visual number-judgment task (Experiment 1) and a vibrotactile secondary task (Experiments 2 and 3).

The analysis revealed a significant effect of *acoustic condition* on memory performance (χ^2^(2) = 35.35, *p* < .001). Pairwise comparisons for *acoustic condition* at each level of *secondary task* separately indicated that more memory errors were made in the single listening task when performed in moderate-noise conditions compared to soft-noise conditions (*z*-ratio = 3.23, *p* = 0.004) and compared to quiet conditions (*z*-ratio = 3.77, *p* < .001). A similar pattern of results emerged for the listening task performed in parallel with the vibrotactile secondary task: participants made more errors in moderate-noise conditions compared to soft-noise conditions (*z*-ratio = 2.55, *p* = 0.03) and compared to quiet conditions (*z*-ratio = 5.05, *p* < .001).

These findings suggest that memory performance in the listening task deteriorates as noise levels increase. Specifically, moderate background noise leads to more errors compared to soft background noise and quiet conditions. This is the case when the listening task is performed in single-task conditions and in dual-task conditions with the vibrotactile task designated as a secondary task.

Furthermore, the effect of *number of tasks* on memory performance was significant (χ^2^(1) = 46.80, *p* < .001). According to the GLMM output participants performance in the listening task was worse in dual-task conditions than in single-task conditions (*z*-ratio = -5.66, *p* < .001).

The effects of *secondary task* on memory performance (χ^2^(1) = 1.11, *p* = 0.29), the interaction between *acoustic condition* x *number of tasks* (χ^2^(2) = 0.34, *p* = 0.85), and the interaction between *acoustic condition* x *secondary task* (χ^2^(1) = 2.57, *p* = 0.11) were not significant.

#### Performance in the secondary tasks

**Fig 4:**
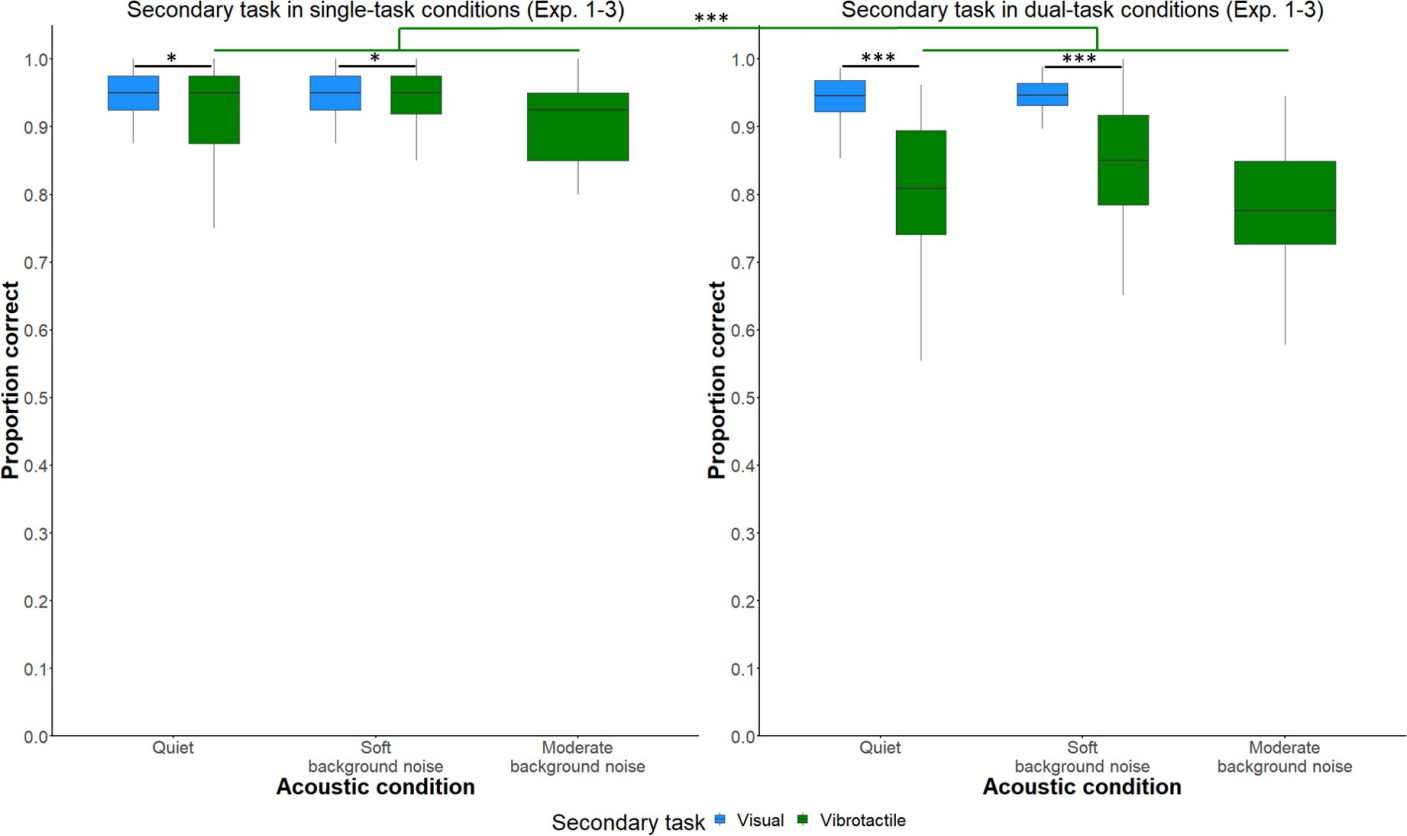
Performance in the secondary tasks across Experiments 1–3. Secondary task performance as a function of *acoustic condition* (quiet, soft background noise, moderate background noise) and *secondary task* (visual number-judgment task, vibrotactile pattern recognition task). Left: Performance in the secondary tasks in single-task conditions. Right: Performance in the secondary tasks in dual-task conditions. The boxplots show the data distributions for secondary task performance (proportion correct). The boxes represent the interquartile ranges, while the lines contained within the boxes represent the medians. ***p < .001, *p < .05.

Next, we investigated the effect of *acoustic condition* and *secondary task* on listening effort based on participants’ secondary task performance. The best-fitting GLMM modeling secondary task performance included *acoustic conditions* (quiet in Experiments 1–3, soft background noise in Experiments 1 and 2, or moderate background noise in Experiment 3), *secondary task* (visual secondary task in Experiment 1 or vibrotactile secondary task in Experiments 2 and 3), *number of tasks* (secondary task as a single task or as a dual task), and their interactions as fixed factors. As a random (intercept) factor, *participant* was included. The descriptive results for performance are shown in Fig 4. Table 3 shows the final GLMM results for performance in the secondary tasks.

**Table 3:** Results from the final GLMM modeling performance in the secondary task as predicted by the acoustic condition and secondary task.

Fixed effects	Estimate	*SE*	*z*	95% CI	*p*
Intercept	2.96	0.14	20.40	14.58, 25.78	< .001
Acoustic condition^a^					
Quiet	Reference				
Soft background noise	-0.01	0.15	-0.08	0.74, 1.32	0.94
Moderate background noise	-0.07	0.11	-0.69	0.75, 1.14	0.49
Soft background noise	Reference				
Moderate background noise	-0.07	0.14	-0.49	0.71, 1.23	0.63
Secondary task^b^					
Visual number-judgment task	Reference				
Vibrotactile pattern recognition task	-0.44	0.18	-2.47	0.46, 0.91	0.01
Number of tasks					
Single-task condition	Reference				
Dual-task condition	-0.09	0.11	-0.80	0.73, 1.14	0.42
Acoustic condition^a^ x Number of tasks					
Quiet x Single task	Reference				
Soft background noise x Dual task	0.06	0.16	0.36	0.77, 1.45	0.72
Moderate background noise x Dual task	0.08	0.12	0.66	0.86, 1.36	0.51
Quiet x Dual task	Reference				
Soft background noise x Single task	-0.06	0.16	-0.36	0.69, 1.29	0.72
Moderate background noise x Single task	-0.08	0.12	-0.66	0.74, 1.16	0.51
Soft background noise x Single task	Reference				
Moderate background noise x Dual task	0.02	0.14	0.11	0.76, 1.35	0.92
Soft background noise x Dual task	Reference				
Moderate background noise x Single task	-0.02	0.14	-0.11	0.74, 1.31	0.92
Acoustic condition^a^ x Secondary task^b^					
Quiet x Visual number-judgment task	Reference				
Soft background noise x Vibrotactile pattern recognition task	0.005	0.19	0.03	0.69, 1.47	0.98
Quiet x Vibrotactile secondary task	Reference				
Soft background noise x Visual secondary task	-0.01	0.19	-0.03	0.68, 1.45	0.98
Secondary task^b^ x Number of tasks					
Visual number-judgment task x Single task	Reference				
Vibrotactile pattern recognition task x Dual task	-0.92	0.13	-6.89	0.31, 0.52	< .001
Visual number-judgment task x Dual task	Reference				
Vibrotactile pattern recognition task x Single task	0.91	0.13	6.86	1.93, 3.26	< .001
Acoustic condition^a^ x Secondary task^b^ x Number of tasks					
Single task x Quiet x Visual number-judgment task	Reference				
Dual task x Soft background noise x Vibrotactile pattern recognition task	0.01	0.21	0.03	0.67, 1.51	0.98
Single task x Quiet x Vibrotactile pattern recognition task	Reference				
Dual task x Soft background noise x Visual number-judgment task	-0.01	0.21	-0.03	0.66, 1.49	0.98
Single task x Soft background noise x Visual number-judgment task	Reference				
Dual task x Quiet x Vibrotactile pattern recognition task	-0.01	0.21	-0.03	0.66, 1.50	0.98
Single task x Soft background noise x Vibrotactile pattern recognition task	Reference				
Dual task x Quiet x Visual number-judgment task	0.01	0.21	0.03	0.67, 1.51	0.98

*Note*. Number of observations: 46939; groups: Participant = 117. Confidence intervals were calculated using the Wald method. Model equation: Correctness ~ acoustic condition*secondary task*number of tasks + (1|participant); family = binomial, link function = logit.

^a^ The primary listening task was presented in quiet (Experiments 1–3), in soft broadband noise (Experiments 1 and 2), and in moderate broadband noise (Experiment 3).

^b^ The secondary task was a visual number-judgment task (Experiment 1) and a vibrotactile secondary task (Experiments 2 and 3).

The GLMM analysis indicated that *secondary task* had a significant effect on performance in the secondary tasks (χ^2^(1) = 88.10, *p* < .001). The GLMM output shows that fewer errors were made in the visual number-judgment task compared to the vibrotactile pattern recognition task (*z*-ratio = -2.47, *p* = 0.01).

Moreover, the interaction of *secondary task x number of tasks* on performance in the secondary tasks was significant (χ^2^(1) = 80.77, *p* < .001). Pairwise comparisons for *secondary task x number of tasks* at each level of *acoustic conditions* separately revealed the following patterns: participants made fewer errors in the visual secondary task in single-task conditions than in the vibrotactile pattern-recognition task in dual-task conditions in quiet (*z*-ratio = -8.71, *p* < .001) and in soft background noise (*z*-ratio = -8.04, *p* < .001). Participants made fewer errors in the visual secondary task in dual-task conditions than in the vibrotactile secondary task in dual-task conditions in quiet (*z*-ratio = -10.08, *p* < .001) and in soft background noise (*z*-ratio = -9.51, *p* < .001). Finally, participants made fewer errors in the vibrotactile secondary task in single-task conditions than in the vibrotactile secondary task in dual-task conditions in quiet (*z*-ratio = -14.06, *p* < .001), in soft background noise (*z*-ratio = -8.53, *p* < .001), and in moderate background noise (*z*-ratio = -10.09, *p* < .001). As indicated by the outcome variable performance, the visual secondary task was less demanding than the vibrotactile secondary task.

#### RTs in the secondary tasks

**Fig 5:**
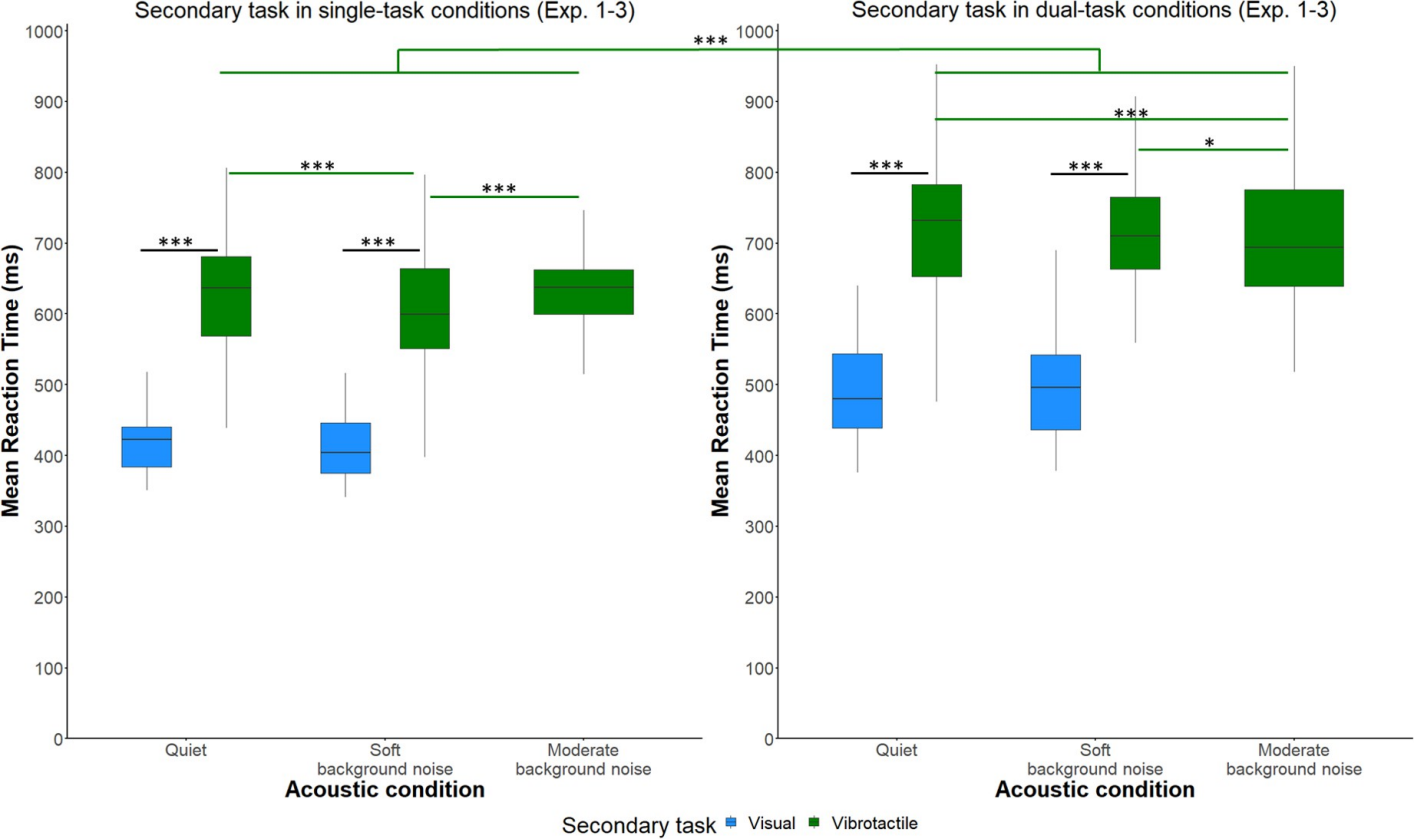
Reaction times in milliseconds in the secondary tasks across Experiments 1–3. Secondary task reaction times as a function of *acoustic condition* (quiet, soft background noise, moderate background noise) and *secondary task* (visual number-judgment task, vibrotactile pattern recognition task). Left: Reaction times in the secondary tasks in single-task conditions. Right: Reaction times in the secondary task in dual-task conditions. The boxplots illustrate the data distributions for the secondary task reaction times. The boxes represent the interquartile ranges, while the lines contained within the boxes represent the medians. ***p < .001, *p < .05.

Finally, we investigated the effect of *acoustic condition* and *secondary task* on listening effort based on participants’ RTs in the secondary tasks. The best-fitting GLMM modeling RTs included *acoustic conditions* (quiet in Experiments 1–3, soft background noise in Experiments 1 and 2), or moderate background noise in Experiment 3), *secondary task* (visual secondary task in Experiment 1 or vibrotactile secondary task in Experiments 2 and 3), *number of tasks* (secondary task as a single task or as a dual task), and their interactions as fixed factors. As a random (intercept) factor, *participant* was included. The descriptive results for RTs are shown in Fig 5. Table 4 presents the final GLMM results for the RTs in the secondary task.

**Table 4:** Results from the final GLMM modeling reaction times in the secondary task as predicted by the acoustic condition and secondary task.

Fixed effects	Estimate	*SE*	*z*	95% CI	*P*
Intercept	6.04	0.03	221.67	396.90, 441.62	< .001
Acoustic condition^a^					
Quiet	Reference				
Soft background noise	-0.01	0.01	-1.32	0.97, 1.01	0.19
Moderate background noise	0.01	0.01	1.09	0.99, 1.03	0.28
Soft background noise	Reference				
Moderate background noise	0.04	0.11	3.67	1.02, 1.07	< .001
Secondary task^b^					
Visual number-judgment task	Reference				
Vibrotactile pattern recognition task	0.40	0.03	12.41	1.40, 1.59	< .001
Number of tasks					
Single-task condition	Reference				
Dual-task condition	0.15	0.01	21.26	1.15, 1.18	< .001
Acoustic condition^a^ x Number of tasks					
Quiet x Single task	Reference				
Soft background noise x dual task	0.02	0.01	1.59	0.99, 1.04	0.11
Moderate background noise x dual task	-0.04	0.01	-3.63	0.94, 0.98	< .001
Quiet x Dual task	Reference				
Soft background noise x Single task	-0.02	0.01	-1.59	0.96, 1.00	0.11
Moderate background noise x Single task	0.04	0.01	-3.63	1.02, 1.06	< .001
Soft background noise x Single task	Reference				
Moderate background noise x Dual task	-0.07	0.01	-5.70	0.91, 0.95	< .001
Soft background noise x Dual task	Reference				
Moderate background noise x Single task	0.07	0.01	5.70	1.05, 1.10	< .001
Acoustic condition^a^ x Secondary task^b^					
Quiet x Visual number-judgment task	Reference				
Soft background noise x Vibrotactile pattern recognition task	-0.02	0.01	-1.53	0.95, 1.01	0.13
Quiet x Vibrotactile secondary task	Reference				
Soft background noise x Visual secondary task	0.02	0.01	1.53	0.99, 1.05	0.13
Secondary task^b^ x Number of tasks					
Visual number-judgment task x Single task	Reference				
Vibrotactile pattern recognition task x Dual task	-0.01	0.01	-1.37	0.97, 1.01	0.17
Visual number-judgment task x Dual task	Reference				
Vibrotactile pattern recognition task x Single task	0.01	0.01	1.37	0.99, 1.03	0.17
Acoustic condition^a^ x Secondary task^b^ x Number of tasks					
Single task x Quiet x Visual number-judgment task	Reference				
Dual task x Soft background noise x Vibrotactile pattern recognition task	0.02	0.01	1.05	0.99, 1.05	0.29
Single task x Quiet x Vibrotactile pattern recognition task	Reference				
Dual task x Soft background noise x Visual number-judgment task	-0.02	0.01	-1.05	0.96, 1.01	0.29
Single task x Soft background noise x Visual number-judgment task	Reference				
Dual task x Quiet x Vibrotactile pattern recognition task	-0.02	0.01	-1.05	0.96, 1.01	0.29
Single task x Soft background noise x Vibrotactile pattern recognition task	Reference				
Dual task x Quiet x Visual number-judgment task	0.02	0.01	1.05	0.99, 1.05	0.29

*Note*. Number of observations: 39626; groups: Participant = 117. Confidence intervals were calculated using the Wald method. Model equation: Correctness ~ acoustic condition*secondary task*number of tasks + (1|participant); family = Gamma, link function = log.

^a^ The primary listening task was presented in quiet (Experiments 1–3), in soft broadband noise (Experiments 1 and 2), and in moderate broadband noise (Experiment 3).

^b^ The secondary task was a visual number-judgment task (Experiment 1) and a vibrotactile secondary task (Experiments 2 and 3).

*Acoustic condition* had a significant effect on RTs in the secondary task (χ^2^(2) = 9.23, *p* = 0.01). RTs in the secondary task were significantly higher in moderate background noise compared to soft background noise (*z*-ratio = 3.67, *p* < .001).

Moreover, the results indicated significant effects of *secondary task* on RTs in the secondary task (χ^2^(1) = 153.31, *p* < .001). According to the GLMM output, RTs were higher in the vibrotactile pattern recognition task than the visual number-judgment task (*z*-ratio = 12.41, *p* < .001), suggesting that the visual number-judgment task was less demanding than the vibrotactile pattern recognition task.

Furthermore, *number of tasks* had a significant effect on RTs in the secondary task (χ^2^(1) = 2007.79, *p* < .001), which were higher in dual-task conditions than in single-task conditions according to the GLMM output (*z*-ratio = 21.26, *p* < .001).

Finally, the interaction of *acoustic condition x number of tasks* significantly influenced RTs in the secondary tasks (χ^2^(2) = 34.15, *p* < .001). Therefore, pairwise comparisons for *acoustic condition x number of* tasks were conducted at both levels of *secondary task* separately. For the visual secondary task, RTs were lower under the following conditions: in quiet single-task conditions than in quiet dual-task conditions (*z*-ratio = -21.26, *p* < .001) and in soft background noise single-task conditions than in soft background noise dual-task conditions (*z*-ratio = -23.42, *p* < .001). For the vibrotactile pattern recognition task, the following results emerged: in single-task conditions, RTs were lower in soft background noise compared to both moderate background noise (*z*-ratio = -3.67, *p* = .003) and quiet conditions (*z*-ratio = -3.49, *p* < .001). In dual-task conditions, a different pattern of results emerged, showing lower RTs in moderate background noise compared to both soft background noise (*z*-ratio = -3.12, *p* = 0.01) and quiet conditions (*z*-ratio = -4.39, *p* < .001). Additionally, RTs were significantly lower in single-task conditions compared to dual-task conditions across all noise conditions: that is, in quiet (*z*-ratio = -22.99, *p* < .001), in soft background noise (*z*-ratio = -19.66, *p* < .001), and in moderate background noise (*z*-ratio = -11.87, *p* < .001).

## Discussion

In this study, three experiments were conducted to investigate the impact of listening conditions in quiet versus listening conditions in soft or moderate broadband noise, where speech remains highly intelligible, on the memory of listeners for conversations between two talkers and on listening effort. In all experiments, we measured memory for running speech by asking participants to answer content-related questions immediately after having heard a two-talker conversation. This was done in a quiet listening condition, as well as in a soft-noise condition (SNR of +10 dB) in Experiments 1 and 2, and a moderate-noise condition (SNR of -3 dB) in Experiment 3. Listening effort was indirectly measured using a dual-task paradigm where this primary listening task was accompanied by an unrelated secondary task. Experiment 1 used a visual number-judgment task, which has been successfully employed in previous studies [9,13,27]. In Experiments 2 and 3, we employed a vibrotactile pattern recognition task as a more demanding secondary task.

First, we investigated the impact of listening conditions in quiet versus soft or moderate broadband noise on memory performance during the HTR task, both independently and concurrently with a secondary task, across all three experiments. The findings clearly demonstrate that moderate background noise (SNR of -3 dB) significantly impairs memory performance during the listening task, both in single-task conditions and dual-task conditions, compared to soft noise (SNR of +10 dB) or quiet conditions. Participants made more memory errors in the HTR when exposed to moderate broadband noise compared to both soft noise and quiet conditions. This deterioration underscores the disruptive impact of moderate noise on cognitive processes involved in memory retention during auditory tasks. The presence of soft background noise, however, did not affect the amount of information that listeners could recall: participants did not remember fewer facts when listening to conversations with soft background noise compared to quiet conditions.

Secondly, we investigated the effects of quiet versus soft or moderate broadband noise on secondary task performance and RTs during the visual number-judgment task and the vibrotactile pattern-recognition task across all three experiments. The findings for performance in these secondary tasks suggest that performance in the secondary task remained consistent across all acoustic conditions in single- and dual-task conditions. However, the findings for RTs were unexpected: participants exhibited faster RTs in the vibrotactile secondary tasks in dual-task conditions performed under moderate compared to soft noise conditions and compared to quiet conditions. This may suggest that, in our study, increased noise level did not increase RTs. This could imply that, under certain conditions, moderate broadband noise might enhance participants’ responsiveness, which could potentially be due to heightened arousal or increased attentional focus as a type of compensation for environmental distractions. Although noise might affect response speed, it did not compromise the performance on the secondary task. This finding shows that participants were able to adapt their cognitive strategies or allocation of resources effectively to maintain performance standards across varying noise environments.

As described in the introduction, the dual-task paradigm can be used to compare different listening conditions, with certain resulting performance patterns serving as indicators of differences in listening effort. A typical explanation of listening effort is a decline in secondary task performance in one experimental condition, while the performance in the primary listening task remains the same in both conditions. However, it is noteworthy that our results are not based on differences in performance or RT in the secondary task under different experimental conditions, but on performance in the primary listening task. The aforementioned results are one example of a pattern indicative of listening effort. We argue that the increase in error rates in the primary listening task during moderate noise compared to quiet conditions in Experiment 3 cannot be interpreted other than in terms of increased listening effort, since speech intelligibility remained high. An increased demand for processing and cognitive resources would result in fewer resources being available to the listener for correctly memorizing the conversation. Regarding the dependent variable RT, we found a different effect of quiet compared to soft and moderate noise conditions for the vibrotactile pattern-recognition task. Participants responded faster in dual-task conditions as the noise level increased, while their performance was unaffected. This is in line with findings from Fintor et al. [9], who also did not observe differences between experimental conditions in performance in the secondary task in two quiet conditions, where the two talkers were spatially separated or co-located.

The results expand on Surprenant’s findings [8]. It was demonstrated that serial recall of unrelated syllables can be impaired in short-term memory tasks, even when speech signals are highly intelligible under different levels of noise (broadband noise presented at either +5 dB SNR or +10 dB SNR) [1]. In our research, this is not the case for our soft-noise condition with a +10 dB SNR and short-term memory of conversational content. It is only true for the moderate-noise condition of -3 dB SNR.

Referring back, our results resonate with the ELU model [23]. Following this model’s assumptions, we would argue that listening in moderate noise with a low SNR activates the explicit cognitive processing route. This route is slower, more focused, and more resource-intensive, leaving fewer cognitive resources available for other cognitive tasks. This leads to fewer correct answers in the primary listening task and a reduced capacity to effectively manage tasks in a dual-task environment.

The vibrotactile pattern-recognition task was more demanding than the visual number judgment task, as indicated by the significantly higher percentage of errors in single-task conditions. It also had more errors when performed in parallel with the listening task (dual-task setting). In addition, the vibrotactile pattern-recognition task is primarily designed to assess tactile processing mostly independently of both the visual and auditory modalities, with headphones used to minimize any auditory interference. This makes this task suitable for studies with speech presented audiovisually. Recently, there has been a growing interest in investigating the impact of visual cues, e.g., gestures and lip movement, on listening effort and short-term memory [25,26]. However, the role of visual cues on listening performance cannot be studied appropriately when the secondary task in the dual-task paradigm is a visual task, i.e., it shares the same modality. For example, Picou and Ricketts [26] faced the problem that to investigate the effect of visual cues on listening effort, the visual probes (secondary task) could not be presented simultaneously with word presentation (primary task). If the visual probes appeared while the talker’s face was moving, the investigated visual cues may have been disrupted, resulting in increased reaction times due to visual distraction. Therefore, a secondary task that is independent of visual and auditory modalities, such as the vibrotactile pattern-recognition task [15,19], allows researchers to explore the role of speech- or conversation-related visual cues in a listening task.

### Limitations

While our study provides valuable insights into the effects of noise on memory performance and listening effort, there are three potential limitations we would like to draw the reader’s attention to.

One potential limitation of our study is the speech intelligibility test. In Experiment 3, a pre-test was conducted to determine how low the SNR could be set while still ensuring that the speech is highly intelligible. However, in the actual experiment, speech intelligibility was not tested for each participant, but instead, participants were queried afterward to ascertain their perceived level of intelligibility. In addition, we conducted hearing screenings to ensure normal hearing sensitivity in both ears. To make sure that future studies build upon our findings, we would recommend considering employing a more comprehensive speech intelligibility test during experiments.

Another potential limitation of our study is the wide age range of participants, particularly in Experiment 1 (18–62 years). The majority of participants were students, with a smaller subgroup of older individuals. Age-related effects on cognitively demanding tasks may introduce variance, necessitating further investigation. However, in our GLMM model for memory performance, we included age as a random (intercept) factor to account for variability across different levels of age. In both GLMM models for secondary task performance, age was not included as it does not explain additional variance beyond that already explained by the other factors in the model. For the reader’s information, scatterplots of performance against age are provided in the S1 Appendix to offer a visual representation of the data and to transparently show the age distribution.

A further limitation of this study is the potential influence of unintended auditory cues from the vibration motors, which were used in the vibrotactile secondary task. While the motors were not audible under continuous broadband noise and auditory stimuli, they may have introduced faint sounds in the quiet single-task conditions. These additional auditory cues could have influenced participants’ RTs in the vibrotactile task, potentially contributing to faster responses in noisy environments compared to quiet conditions. Therefore, we recommend that future researchers carefully consider and control for the potential auditory effects of vibration motors when designing experiments.

## Conclusion

To summarize, the present study revealed that more cognitive resources are consumed in moderate noise conditions (where the SNR is low, but speech is highly intelligible) than in soft noise or quiet conditions, as evidenced by lower performance in the primary listening task. Thus, short-term memory of conversations or other cognitive tasks can be affected.

In recent decades, research on listening has primarily focused on simple cognitive tasks in challenging listening conditions. However, it may now be appropriate for listening research to incorporate more complex cognitive listening tasks, such as short-term memory of conversations. Our study adds to a growing body of research that conducts auditory research in more plausible settings, both in terms of the primary listening task and the listening environment.

## Supporting information

S1 Dataset Processed listening task data of Experiments 1–3. (XLSX)

S2 Dataset Processed secondary task data of Experiments 1–3. (XLSX)

S1 Appendix Age effect on performance. Scatterplots of age against performance.
(PDF)
