# Supplementary material for: Exploring short-term memory and listening effort in two-talker conversations: The influence of soft and moderate background noise
Source: PLoS One. 2025 Feb 6;20(2):e0318821. doi: 10.1371/journal.pone.0318821 (PMC11801578; doi:10.1371/journal.pone.0318821)

S3 Appendix. Age effect on performance.

Fig S3a. Performance in the primary listening task across Experiments 1-3 against age.

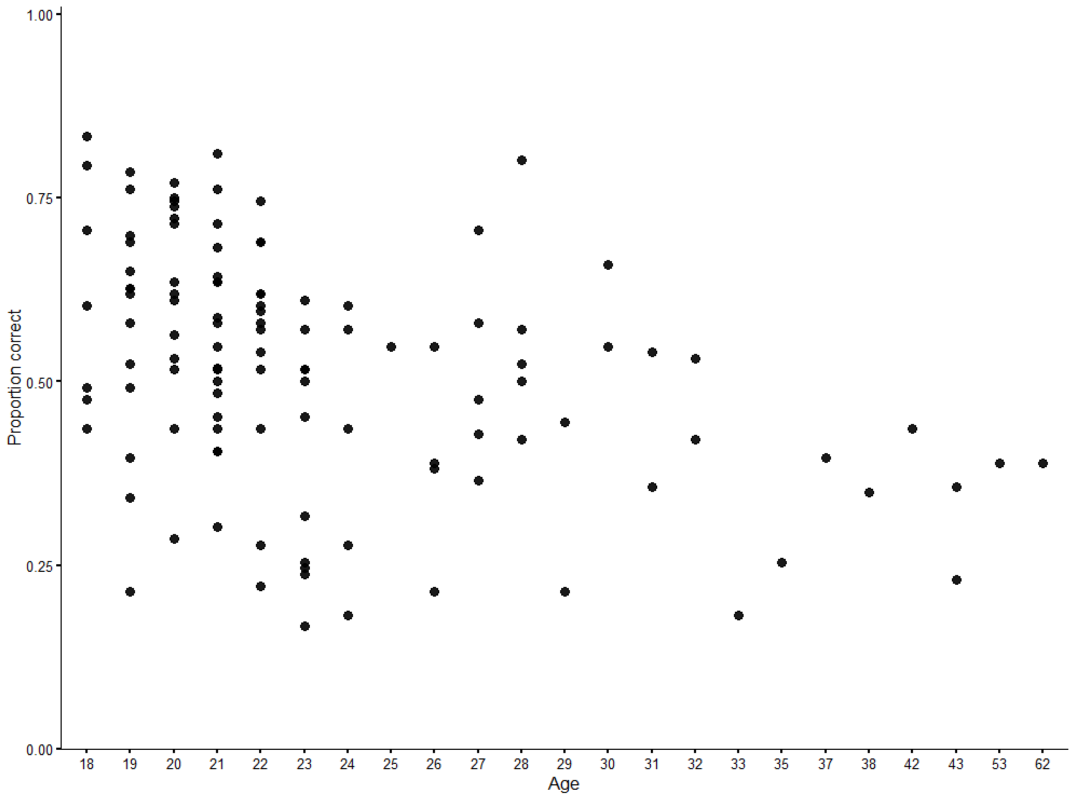

Fig S3b. Performance in the secondary tasks across Experiments 1-3 against age.

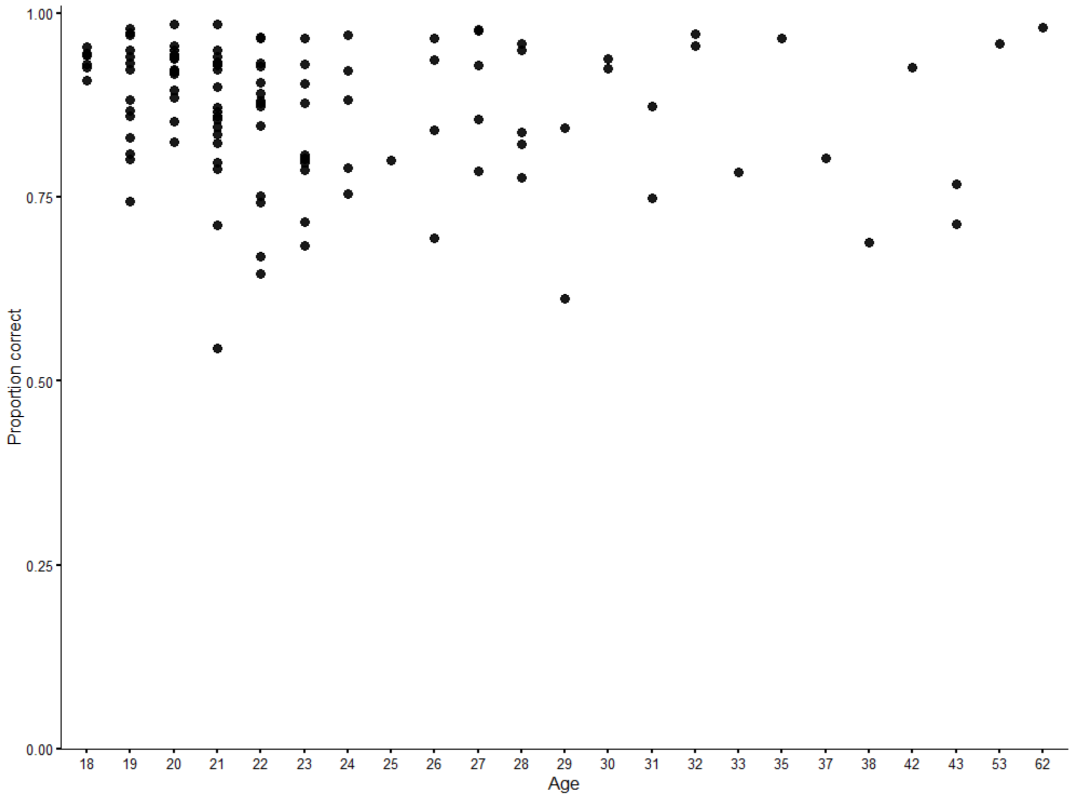

Fig S3c. RTs in the secondary tasks across Experiments 1-3 against age.

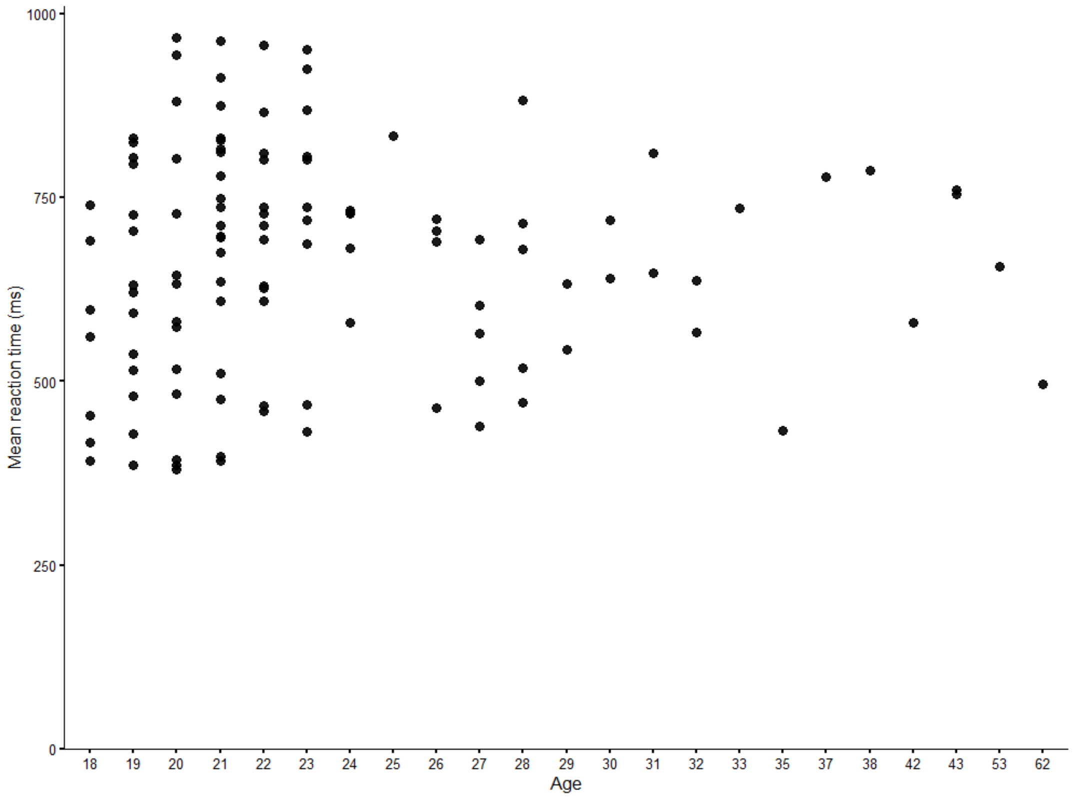

Supplement: S1 Appendix — Scatterplots of age against performance. (PDF) [file pone.0318821.s003.pdf]
